# Platelet-rich plasma versus lidocaine as tenotomy adjuvants in people with elbow epicondylopathy: a randomized controlled trial

**DOI:** 10.1186/s13018-019-1153-6

**Published:** 2019-04-23

**Authors:** Jose Ignacio Martin, Leire Atilano, Josu Merino, Igor Gonzalez, Gotzon Iglesias, Luis Areizaga, Paola Bully, Gonzalo Grandes, Isabel Andia

**Affiliations:** 1 Interventional Sonography, Department of Radiology, Cruces University Hospital, Barakaldo, Spain; 2 Regenerative Medicine, BioCruces Health Research Institute, Cruces University Hospital, 48903 Barakaldo, Spain; 3 Department of Orthopaedic Surgery, Cruces University Hospital, Barakaldo, Spain; 4 Primary Care Research Unit of Bizkaia (Basque Healthcare Service), BioCruces Health Research Institute, Bilbao, Spain

**Keywords:** Elbow tendinopathy, Epicondylitis, Lateral, Medial, Tenotomy, Platelet-rich plasma, Function, Pain, Randomized controlled study, Sonography

## Abstract

**Objectives:**

To determine the efficacy of platelet-rich plasma (PRP) compared to lidocaine as a tenotomy adjuvant for people with elbow tendinopathy.

**Methods:**

Our study was a parallel-group, double-blind, randomized trial involving 71 patients with recalcitrant elbow tendinopathy who received two sessions of ultrasound-guided tenotomy with either PRP or lidocaine in a tertiary public hospital. The primary end point was the percentage of patients with an improvement exceeding 25% reduction in disability (Spanish version of the Disabilities of the Arm, Shoulder and Hand questionnaires–DASH-E) at 6 and 12 months; the secondary outcome was the percentage of patients exceeding 25% reduction in pain (VAS-P).

**Results:**

There was no evidence of significant differences in the proportion of patients who experienced clinically relevant improvements. After 6 months, 18 patients (78.59%) in the lidocaine group and 19 patients (73.08%) in the PRP group showed improved function above 25% (unadjusted odds ratio, 0.90; 95% confidence interval [CI], 0.90 (0.17 to 4.60)); 21 patients (72.21%) in the lidocaine group versus 22 patients (84.62%) in the PRP group achieved more than 25% pain reduction (unadjusted odds ratio, 0.48; 95% CI, 0.10 to 2.37). After 12 months, 17 patients (70.83%) in the lidocaine group versus 19 patients (76%) in the PRP group had improved function (unadjusted odds ratio, 0.71; 95% CI, 0.13 to 3.84), and 19 patients (76%) in the lidocaine group versus 20 patients (90.91%) in the PRP group had improved pain above 25% (unadjusted odds ratio, 0.35; 95% CI, 0.06 to 2.51). Hypercholesterolemia and baseline vascularization influenced outcomes. There were no differences between groups in the adjusted odds ratios.

**Conclusion:**

PRP results in similar improvements to those obtained with lidocaine. Selecting patients according to their pretreatment status can improve treatment efficacy.

**Trial registration:**

NCT01945528, EudraCT 2013-000478-32. Registered 18 August 2013, enrolment of the first participant 10 March 2014

## Introduction

Elbow tendinopathy (epicondylalgia) is the most common tendinopathy in upper limbs and it has a relevant economic burden [1]. It can affect the wrist extensors that originate from the lateral epicondyle and/or the flexors originating from the medial epicondyle, with similar rates between men and women. Lateral epicondylopathy is more prevalent than medial epicondylopathy, i.e., 1.3% versus 0.4% [2], but the prevalence could be as high as 5.2% or higher in middle-aged people performing repetitive movements and/or forceful tasks as part of their working activities [3, 4].

Local injection treatments are proposed as a cost-effective conservative management in patients who are recalcitrant to bracing, non-steroidal anti-inflammatory drugs (NSAIDs), or physiotherapy before considering tendon release through arthroscopy or open surgery. The most common injectable is corticosteroids, a palliative treatment frequently administered on the peritendon. Actually, the target could be sympathetic and sensory innervation could be found in the superficial side of the extensor carpi radialis brevis (ECRB) origin [5]. However, in persistent elbow tendinopathy, corticosteroid injections delayed complete recovery and increased the recurrence rate [6]; therefore, research has shifted towards the development of tendon regenerative treatments.

Needle tenotomy is a commonly performed therapy that involves passing a needle through the abnormal tendon multiple times, with intensities that can vary according to the number of passes, ranging from 5 to 50, with needle diameters ranging from 20 to 25 gauge (also described as fenestrations, needling, or peppering) [7]. Any type of tenotomy can be considered a regenerative technique because ensuing microtrauma induces a healing response through an early gene expression (transcription factors) pattern similar to mechanical loading, thereby enhancing tendon structure and strength [8]. The goal is to convert a chronic degenerative process into acute inflammation by breaking the vicious loop of failed healing and remodelling provoked by accumulated tendon damage [9]. Nevertheless, few controlled studies have examined the efficacy of percutaneous needle tenotomy for elbow epicondylopathy [10].

Moreover, tenotomy can be “dry” (as a standalone procedure) or can be part of a combined intervention; i.e., associated with anaesthetics, corticosteroids, or regenerative products, such as blood or platelet-rich plasma (PRP). PRP consists of a plasma preparation with a concentration of platelets above peripheral blood (pure PRP) and optionally with concentrated leukocytes (L-PRP). The molecular pool present in PRP modulates biological processes involved in tissue healing, including modulation of angiogenesis and inflammation as well as cell proliferation and survival [11]. In addition, it provides a fibrin template that can guide cells in the injured areas. Although these biological actions have been shown in vitro [12], the clinical efficacy of PRP preparations in tendons is still controversial.

PRP has been widely used to treat diverse tendinopathies, e.g., rotator cuff, Achilles, patellar and gluteal tendons, but current meta-analyses have shown modest improvements [13–15]. Specifically, the value of PRP in epicondylopathy has been investigated in more than a dozen controlled trials [16], mostly using corticosteroids or whole blood as comparators. However, efficacy has not been demonstrated yet, in part due to heterogeneity among clinical studies that was mainly attributed to variability in PRP formulations [17] and procedures of delivery. In addition, whether PRP is more efficient in enthesopathies, such as epicondylopathy, or tendinopathies in the proper tendon is controversial [18].

Our objective was to examine whether pure PRP (with a moderate concentration of platelets) is a good adjuvant to tenotomy in the management of chronic elbow tendinopathy (medial and lateral). We have used [tenotomy + lidocaine] as a comparator, which is our gold standard. Consistent with the classic view of chronic wounds where a single intervention may be insufficient to regenerate a chronic injury, we performed two interventions with a 2-week interval.

## Methods

We performed a parallel-group, assessor- and patient-blinded, randomized controlled trial in a tertiary public hospital.

Inclusion criteria were tendinopathy present in either the lateral or medial elbow in patients who had failed conservative treatments. The latter consisted of 4–6 weeks of antialgic and anti-inflammatory medication (NSAIDs), physical therapy associated with orthosis and at least one corticosteroid infiltration in the painful area. Patients were included if they had symptoms lasting at least 3 months or longer and baseline elbow pain above 3/10 during resisted wrist extension or flexion, in the case of lateral or medial epicondylopathy respectively. Other inclusion criteria were age between 18 and 75 years, body mass index (BMI) between 20 and 35 and commitment to comply with all study procedures. The most symptomatic elbow was treated in bilateral patients. Patients suspended any analgesic therapy 15 days before the intervention; merely paracetamol 1 gr/6 h or metamizole granulated oral suspension, maximum 4 g/day, was allowed. Oral corticoids were not permitted nor were NSAIDs for up to ten or more consecutive days.

The exclusion criteria were the presence of a full tendon tear; BMI > 35; systemic autoimmune rheumatologic disease (connective tissue diseases and systemic necrotizing vasculitis); poorly controlled diabetes mellitus (glycosylated haemoglobin above 9%); blood disorders (thrombopathy, thrombocytopenia, anaemia with a Hb level < 9); receiving immunosuppressive treatments; receiving local steroid injection within 3 months of randomization; receiving NSAIDs, opioids, or oral corticosteroids within 15 days before inclusion in the study; severe heart disease; patients unable to comply with scheduled visits due to work, or spending long periods away from their habitual residence; patients with active cancer or cancer diagnosed in the last 5 years; analytical diagnosis of hepatitis B or C or HIV infection; pregnant or lactating; and patients who were taking a drug in a clinical investigation. Initial patient selection was conditioned to the negative results in the analytical tests for hepatitis B or C or HIV infection.

The diagnosis was performed by orthopaedic surgeons and was merely clinical. Lateral epicondylopathy was diagnosed based on pain and maximum tenderness upon palpation at the common extensor origin at the lateral humeral epicondyle, pain upon resisted forearm supination, painful active wrist extension and painful passive wrist flexion. Medial epicondylopathy was diagnosed based on pain distal to the common flexor insertion, increased pain with resisted wrist flexion and pain with resisted forearm pronation and elbow extension. Patients with suspected ulnar neuropathy were not included. Clinical assessments were performed by the same orthopaedic surgeons that performed the inclusion assessment and were unaware of the treatment modality.

### Settings and locations

The study protocol was approved by the local Ethics Committee of HUC, authorized by the Spanish Agency of Medicines (EudraCT 2013-000478-32), registered at clinicaltrials.gov (NCT01945528) and was published previously [19].

Between April 2014 and May 2017, a total of 85 patients with elbow tendinopathy were referred to the Orthopaedic Department at Cruces University Hospital, by general practitioners or by other orthopaedic departments, and they were assessed for eligibility. Eighty-two patients met the inclusion/exclusion criteria and underwent blood tests. All patients provided written informed consent and could decide to leave the study at any time.

### Interventions

The experimental and control groups were ultrasound (US)-guided percutaneous tenotomy combined with PRP each alternate week for a total of two interventions and US-guided tenotomy combined with lidocaine each alternate week for a total of two interventions.

### PRP preparation

Twenty-four mL of peripheral blood (i.e., three 9 mL tubes containing 0.9 mL of sodium citrate, Vacuette, Greiner BioOne, Switzerland) were withdrawn from all patients at every intervention. Pure (leukocyte-free) PRP was prepared by single spinning at 570 G for 6 min and the plasma layer was collected, under laminar flow, avoiding aspirating the buffy coat, following our standard operating procedures. In doing so, we obtained approximately 6-8 mL of pure PRP (no leukocytes) without detectable leukocytes and a moderated enrichment of platelets (2.30 ± 0.68 times above peripheral blood baseline). According to previous classifications [20], it can be described as pure platelet-rich plasma (P-PRP) or leukocyte-poor platelet-rich plasma, i.e., preparations without leukocytes and with a low-density fibrin network after activation.

At the interventional radiologist office, PRP is activated with CaCl_2_ (final concentration 22.5 mM) prior to loading 5 mL in a 10-mL Luer-lock syringe.

### Tenotomy

We performed all procedures with the patient in a supine position, the elbow flexed 120° and forearm in pronation (lateral) or supination (medial), guided with a 4-13 MHz high-frequency linear probe (Esaote MyLab 70 XVG, Esaote S.p.A. Genoa, Italy). A senior radiologist with more than 20 years of experience in musculoskeletal interventional ultrasonography performed all tenotomies.

A sterile protocol was followed as for any other musculoskeletal intervention. The subcutaneous tissues overlying the lateral epicondyle were infiltrated tangential to the plane of the lateral epicondyle with 2 mL lidocaine via a 22-gauge hypodermic needle. Then, the bulb containing the injectable (PRP or lidocaine) was connected to the needle, which was inserted parallel to the tendon long axis, from distal to proximal. The tendon was repeatedly fenestrated (15–25 times) by redirecting the needle in different directions, until softening of the tissue. In addition to piercing the tendon, the tip needle was used to abrade the periosteum. At the same time, the injectable was delivered in the areas of hypoechogenicity and the surround. A second intervention, involving approximately ten tendon perforations and no abrasions of the periosteum, was performed after 2 weeks. We injected 4.23 ± 1.09 mL (range 1–5) of lidocaine and 4.47 ± 1.11 (range 1–5) of PRP in the first intervention and 4.18 ± 1.14 mL (range 1–5) of lidocaine and 4.53 ± 0.88 mL (range 2–5) of PRP in the second intervention. There were no differences between the injected volumes. After each intervention, patients were instructed to rest for the first 48 h and avoid weight lifting. Patients did not follow any post-procedural exercise programme, but they modified their activities and resumed physical work upon demand.

### Outcomes

The primary outcome measures were the number (percent) of patients who achieved clinically relevant improvement, defined as a reduction in Disabilities of the Arm, Shoulder and Hand self-reported questionnaires, Spanish version (DASH-E), of at least 25% relative to baseline [21] at 6 and 12 months. Secondary outcome measures were the number (percent) of patients who achieved a clinically relevant improvement in pain, VAS-P (0–10 Likert scale), at 6 and 12 months. For safety assessments, patients recorded any adverse reaction and needed medication in a diary that was collected at each hospital visit. Acetaminophen was the only drug allowed for pain.

### Sample size

We assumed that the relative improvement with the PRP intervention was 1.43, assuming that the differences between PRP and lidocaine would be similar to the differences reported with corticoids [22], and a patient loss of approximately 20%. A sample size of 80 patients was expected to provide an 80% potency to detect any significant difference between the success rates in both groups (P1 = 0.93 and P2 = 0.65) with a level of significance of 5%, with each arm formed by 40 patients.

### Randomization and blinding

An independent researcher performed randomization in blocks of four, using EPIDAT3.1, and created aluminium paper blinded envelopes with the numbered treatment allocation. The numbered envelopes were opened on the treatment day by the researcher who was in charge of the PRP preparation. All physicians (including orthopaedists involved in clinical outcome assessments and radiologists involved in ultrasound assessments), except one radiologist who performed the procedures, were unaware of treatment allocation. All patients were blinded to the treatment. Peripheral blood was drawn from all patients, and in each intervention, the syringe containing the treatment was wrapped with gauze hindering treatment visualization.

### Statistical methods

To test the overall effect of the treatment, [tenotomy + PRP] and [tenotomy + lidocaine], on an intention-to-treat basis, we compared changes in function and pain levels between the two groups over 12 months. No imputation method was used to handle the missing data. To confirm comparability between groups at baseline, Student’s *t* test was used for continuous variables due to the proven normal distribution of the data in both groups, and a *x*^2^ test was used for categorical variables. Spearman’s tests were used to assess the association between outcome variables. Data are summarized as the mean and standard deviation for normally distributed variables, medians and interquartile ranges for non-normally distributed variables and the frequency and percentage for categorical variables. To evaluate the differences between the PRP and lidocaine groups, longitudinal generalized mixed models, with and without adjustments, were used, considering the repeated four follow-up measurements for each patient. The treatment, the time of measurement and treatment-by-time interaction were included as fixed effects in the models. Patients were included as random effects in the intercept of the different repeated measurements. Time evolution was considered as a categorical variable without autocorrelation within an individual. These options were chosen because they provided a better fit to our data. The overall effect of the treatment was assessed by testing the interaction between the treatment and time of measurement. Additionally, these models were also adjusted for baseline values of the outcome variables, socio-demographics and risk factors, possible determinants of pain and function. Likewise, to simplify the fixed effects structure, maximum likelihood ratio tests were used following backward, forward and stepwise strategies. Unadjusted and adjusted odds ratios with 95% confidence intervals (CI) and mean differences with 95% CI were calculated for categorical and continuous outcomes, respectively. Planned contrasts were used to determine whether changes in the PRP group between baseline and each of the follow-up points were different from those observed in the lidocaine group. For all the contrasts, *p* < 0.05 was considered the significance criterion.

A post hoc power calculation was performed based on longitudinal mixed effects models adjusted to the final sample size, actual data variability and clustering to detect differences with small (0.2 standard deviations), medium (0.5 standard deviations) and large (0.8 standard deviations) effect sizes between the comparison groups. All analyses were carried out using the SAS statistical package version 9.4.

## Results

**Fig. 1:**
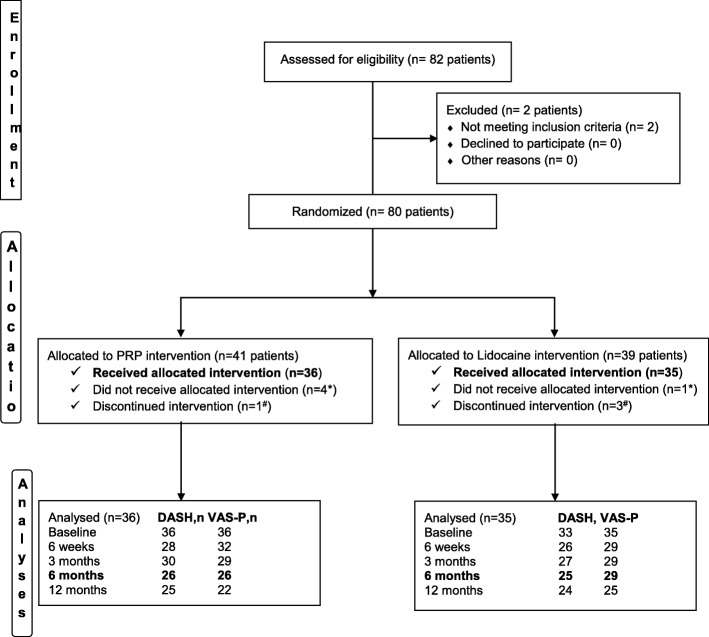
Participants’ flow diagram. Asterisk denotes did not receive allocated intervention because of: a horse accident (1), preferred the surgical option (1), entitled for hip surgery (1), had no pain the day of the first intervention (1) and declined without any reason (1). Number sign indicates discontinued intervention because of post-tenotomy pain. During the study, one patient treated with PRP was lost to follow-up after the 6-week assessment and four patients (three from the lidocaine group and one from the PRP group) were lost after the 6-month assessment. We had additional missing data for two main reasons. First, many patients did not fill the DASH properly (i.e., with less than 25 answers over 30 questions, the DASH was not valid for analyses). Second, some patients attended to the echography service during follow-up but failed orthopaedists ‘consultation where clinical data were collected

Figure 1 represents the flow of participants through the trial. Eighty patients, recruited between 2014 and 2017, meeting all inclusion criteria, were randomly allocated to the [tenotomy + PRP] and [tenotomy + lidocaine] groups. Four patients in the PRP group and one patient in the lidocaine group withdrew from the study before treatment for the following reasons: horse accident (1), preferred the surgical option (1), entitled to hip surgery (1), had no pain the day of the first intervention (1) and declined without any reason (1). One patient in the PRP group and three in the lidocaine group received only the first tenotomy and declined to receive the second intervention because they found the procedure too painful. A total of 71 patients were treated. During the study, one patient treated with PRP was lost to follow-up after the 6-week assessment and four patients (three from the lidocaine group and one from the PRP group) were lost after the 6-month assessment. We had additional missing data for two main reasons. First, many patients did not fill out the DASH-E properly (i.e., less than 25 answers over 30 questions, making the DASH-E invalid for analyses). Second, some patients attended the radiology service during follow-up but failed to complete orthopaedist consultations where clinical data were collected. Sociodemographic and clinical characteristics were well-balanced at baseline (shown in Table 1). The association between pain and function increased throughout the follow-up in a similar way for both groups (see Table 2).

**Table 1:** Baseline sociodemographic clinical and sonographic characteristics in both groups

Variable	Lidocaine (*N* = 35)		PRP (*N* = 36)	
Age, *n*, M ± SD	35	48.26 ± 7.64	36	50.80 ± 6.73
Gender, no. (%)				
Male	19	(54.3)	14	(38.9)
Female	16	(45.7)	22	(61.1)
Manual worker, no. (%)				
No	10	(29.4)	11	(28.2)
Yes	24	(70.6)	25	(71.8)
Throwing sports, no. (%)				
No	29	(87.9)	33	(94.3)
Yes	4	(12.1)	2	(5.7)
Other sports, no. (%)				
No	20	(60.6)	22	(62.9)
Yes	13	(39.4)	13	(37.1)
BMI, *n*, M ± SD	34	26.05 ± 3.15	36	25.58 ± 4.19
Hypercholesterolemia, no. (%)				
No	29	(82.9)	34	(94.4)
Yes	6	(17.1)	2	(5.6)
Hypercholesterolemia treatment				
No	31	(88.6)	35	(97.2)
Yes	4	(11.4)	1	(2.8)
Diabetes, no. (%)				
No	34	(97.1)	36	(100.0)
Yes	1	(2.9)	0	(0.0)
Diabetes treatment				
No	34	(97.1)	36	(100.0)
Yes	1	(2.6)	0	(0.0)
Hyperuricaemia, no. (%)				
No	35	(100.0)	36	(100.0)
Tendon involvement, no. (%)				
Lateral	31	(88.6)	29	(80.6)
Medial	4	(11.4)	7	(19.4)
Location, no. (%)				
Left	9	(25.7)	15	41.7
Right	26	(74.3)	21	58.3
Neovascularization, no. (%)				
No neovascularization	7	(20.0)	6	(16.7)
Neovessels on the tendon surface	2	(5.7)	3	(8.3)
Intratendinous neovessels	26	(74.3)	27	(75.0)
Echotexture grading scale, no. (%)				
Normal	3	(8.6)	1	(2.8)
Hypoechogenicity < 1/3 of the tendon	2	(5.7)	4	(11.1)
Hypoechogenicity > 1/3 and < 2/3	10	(28.6)	11	(30.6)
Hypoechogenicity > 2/3	15	(42.9)	17	(47.2)
Partial-thickness tear	5	(14.3)	3	(8.3)
Calcifications, no. (%)				
No	28	(80.0)	32	(91.4)
Yes	7	(20.0)	3	(8.6)
Tendon thickness, *n*, M ± SD	35	1.16 ± 0.21	36	1.17 ± 0.21
DASH-E, *n*, M ± SD	33	44.06 ± 14.05	36	44.74 ± 17.09
VAS-P, *n*, M ± SD	35	5.87 ± 1.52	35	5.91 ± 1.78

*BMI* body mass index, *DASH-E* Spanish version of the Disabilities of the Arm, Shoulder and Hand questionnaires, *M* mean, *SD* standard deviation, *PRP* platelet-rich plasma, *VAS-P* visual analogue scale for pain\

**Table 2:** Association between DASH-E and VAS-P by treatment

VAS-P	DASH-E				
	Basal	6 weeks	3 months	6 months	12 months
Basal, *r* (*n*)	0.28* (33) 0.52* (35)	0.59* (25) *0.26 (28)*	0.42* (26) *0.28 (27)*	0.46* (25) *0.24 (25)*	0.16 (25) *0.25 (24)*
6 weeks, *r* (*n*)	0.26 (28) *0.34 (32)*	0.70* (24) 0.83* (28)	0.63* (24) 0.67* (25)	0.48*(22) 0.47* (23)	0.37 (24) 0.45* (22)
3 months, *r* (*n*)	0.23 (27) *0.21 (29)*	0.53* (23) 0.60* (24)	0.74* (25) 0.79* (27)	0.63* (23) 0.77* (23)	0.51*(23) 0.76* (20)
6 months, *r* (*n*)	0.34* (27) *0.20 (27)*	0.56* (22) 0.63* (22)	0.67* (23) 0.84* (25)	0.94* (24) 0.88* (23)	0.73* (23) 0.79* (21)
12 months, *r* (*n*)	0.11 (24) *0.07 (23)*	0.32 (21) 0.42*(18)	0.34 (22) 0.50*(21)	0.64* (21) 0.63* (20)	0.91* (24) 0.77* (20)

Data without italics represent lidocaine; data with *italics* represent PRP

*DASH-E* Spanish version of the Disabilities of the Arm, Shoulder and Hand questionnaires, *r* Spearman’s rank correlation coefficient, *VAS-P* visual analogue scale for pain assessment

*p < 0.05

Boldface and bold italics represent statistically significative associations

### Primary and secondary clinical outcomes

Successful treatment was defined as more than 25% reduction in DASH-E or VAS-P scores after 1 year. Table 3 shows the rate of patients who met these criteria in each measurement. A high rate of patients showed enhanced function and pain reduction over the 12-month follow-up.

**Table 3:** Outcome measurements and adjusted intergroup differences

	Unadjusted change			Odds ratio (95% CI)	
Outcome	Lidocaine	PRP	Treatment-time measurement interaction (*p* value)	Unadjusted	Adjusted^a^
% meeting DASH-E improvement, no./total no. (%)			.307		
6–7 weeks	11/24 (45.83)	10/28 (35.71)		1.37 (0.31 to 6.04)	1.33 (0.31 to 5.71)
3 months	20/24 (83.33)	17/29 (58.62)		4.73 (0.86 to 26.08)	5.67 (0.98 to 32.74)
6 months	18/25 (72.00)	19/26 (73.08)		0.90 (0.17 to 4.60)	1.15 (0.22 to 5.95)
12 months	17/24 (70.83)	19/25 (76.00)		0.71 (0.13 to 3.84)	0.91 (0.17 to 4.88)
% meeting VAS-P improvement, no./total no. (%)			.105		
6–7 weeks	19/30 (63.33)	13/31 (41.94)		2.42 (0.67 to 8.76)	2.67 (0.74 to 9.68)
3 months	22/29 (75.86)	16/28 (57.14)		2.78 (0.68 to 11.38)	2.92 (0.71 to 12.04)
6 months	21/29 (72.41)	22/26 (84.62)		0.48 (0.10 to 2.37)	0.50 (0.10 to 2.48)
12 months	19/25 (76.00)	20/22 (90.91)		0.35 80.06 to 2.51)	0.42 (0.06 to 3.03)
Median DASH-E scores (IQR)			.429		
6-7 weeks	30.17 (30.0)	40.59 (34.86)		NA	NA
3 months	16.49 (29.04)	31.73 (34.94)		NA	NA
6 months	12.93 (28.44)	14.25 (26.66)		NA	NA
12 months	7.50 (31.15)	9.17 (24.17)		NA	NA
Median VAS-P scores (IQR)			.441		
6–7 weeks	4.00 (3.00)	4.25 (4.75)		NA	NA
3 months	2.00 (2.50)	4.00 (5.00)		NA	NA
6 months	2.00 (4.40)	2.00 (3.50)		NA	NA
12 months	2.00 (3.50)	2.00 (4.00)		NA	NA

*CI* confidence interval, *DASH-E* Spanish version of the Disabilities of the Arm, Shoulder and Hand questionnaires, *IQR* interquartile range, *NA* not applicable, *no* number, *VAS-P* visual analogue scale for pain assessment

^a^% meeting DASH-E and VAS-P improvement were adjusted for the degree of vascularization before treatment

Data for functional improvement were available for 25 (71.43%) and 24 (68.57%) patients in the lidocaine group and 26 (72.22%) and 25 (69.44%) patients in the PRP group, at 6 and 12 months, respectively. There were no significant differences between therapies in terms of the rate of patients who achieved the minimum clinically important difference in function recovery at 6 or 12 months after treatment (Table 3). The differences in the percentage of success between [tenotomy+lidocaine] and the [tenotomy+PRP] were − 1.94% (95% CI, − 30.46 to 26.59) at 6 months and − 6.03% (95% CI, − 35.98 to 23.90) at 12 months.

In the case of pain, the frequencies for the available data were 29 (82.85%) and 25 (71.43%) in the lidocaine group and 26 (72.22%) and 22 (61.11%) in the PRP group. Likewise, there were no differences between tenotomy adjuvants (lidocaine versus PRP) in the rate of patients who achieved clinically important pain relief and differences in the percentage of success at 6 months, − 10.56% (95% CI, − 33.01 to 11.89), and at 12 months, − 11.51% (95% CI, − 32.29 to 9.27). The differences between the two groups in the short term were not statistically significant either. The difference in the percentage of patients with better functionality at 6 weeks was 7.53% (95% CI, − 27.62 to 42.67) and at 3 months was 26.62% (95% CI, − 0.82 to 54.06). The difference in the percentage of patients with pain reduction exceeding 25% at 6 weeks was 21.70% (95% CI, − 8.63 to 52.02) and 20.81% (95% CI, − 6.84 to 48.47) at 3 months.

**Fig. 2:**
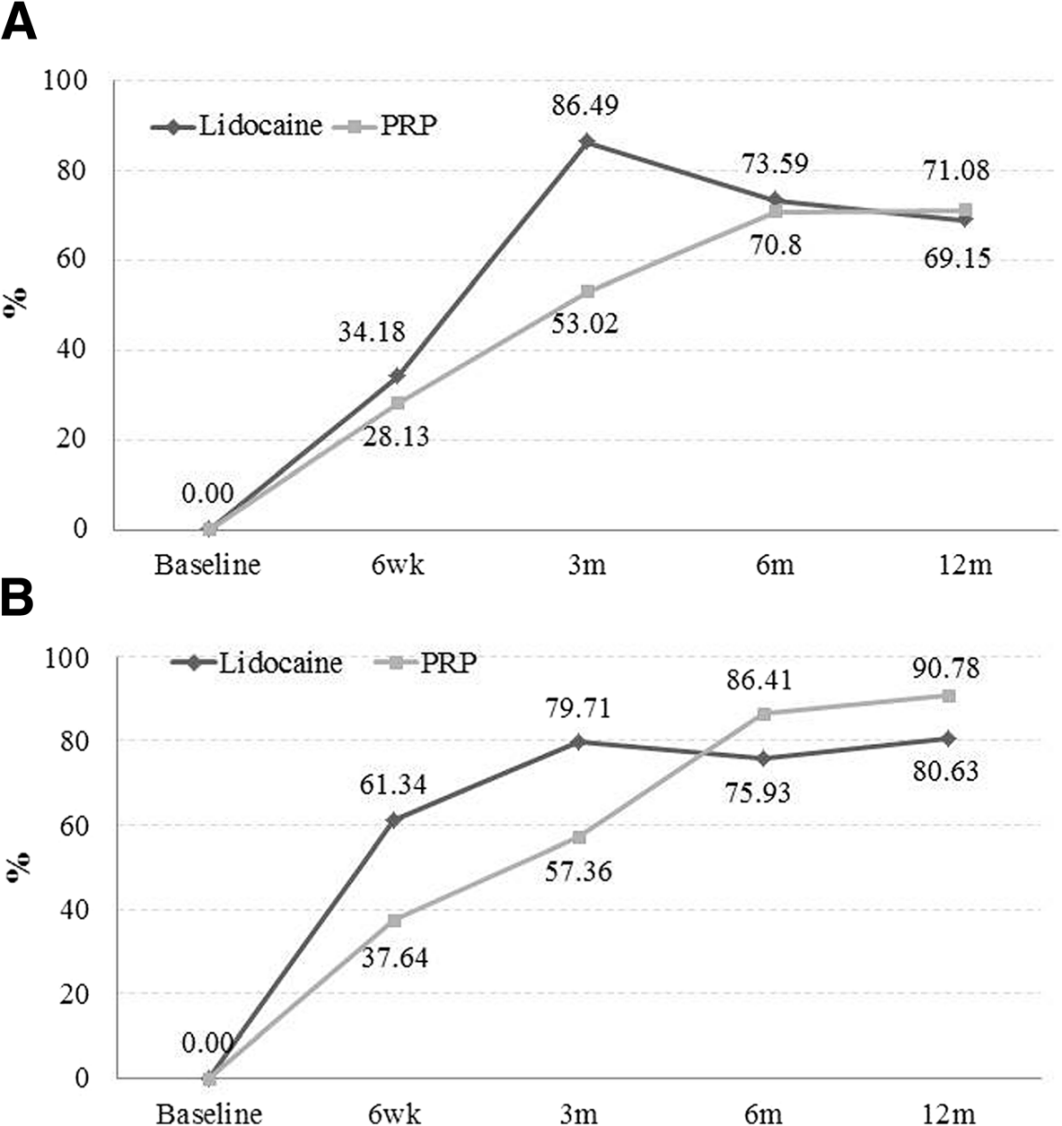
Percentage of patients with an improvement exceeding 25% reduction in DASH-E (**a**) and in VAS-P (**b**) over 1-year follow-up, after adjusting data for baseline vascularization. DASH-E, Spanish version of the Disabilities of the Arm, Shoulder and Hand questionnaires; VAS-P, visual analogue scale for pain; m, month

Essentially, the time course was different between treatments. In the PRP group, the increase in the rates of patients who achieved functional improvement and pain reduction was steadier than in the lidocaine group, which reached a plateau at 3 months. Furthermore, there was no statistical evidence of a modification in treatment effects by the controlled patient’s factors. When data were adjusted for confounders, both groups showed enhanced function and pain reduction over the 12-month follow-up without significant differences between PRP and lidocaine (see Fig. 2 and Table 3).

**Fig. 3:**
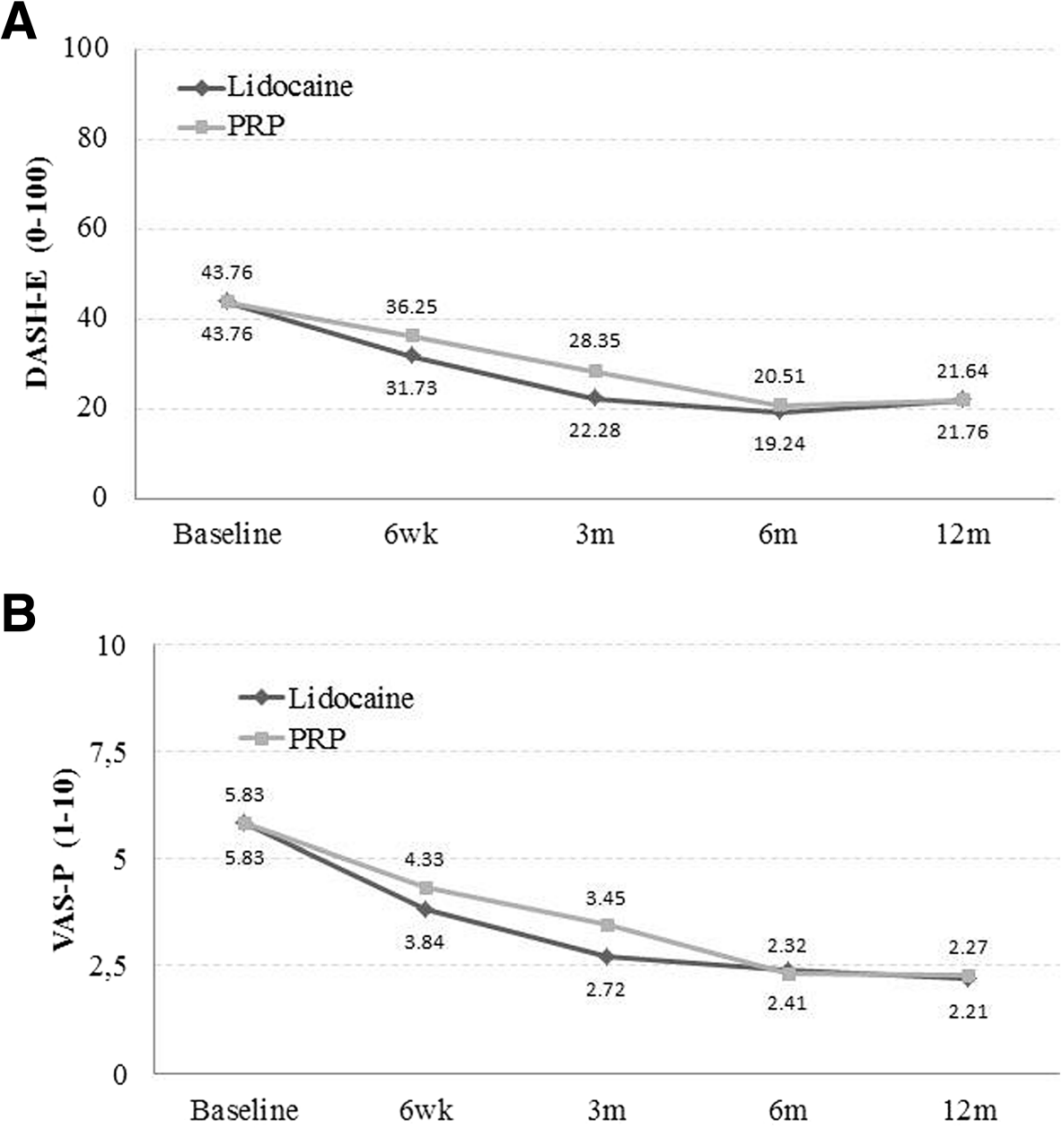
Changes of DASH-E (**a**) and VAS-P (**b**) over time, after adjusting data for baseline DASH-E and VAS-P scores, vascular status and hypercholesterolemia. DASH-E, Spanish version of the Disabilities of the Arm, Shoulder and Hand questionnaires; PRP, platelet-rich plasma; VAS-P, visual analogue scale for pain; m, month

There were no differences between lidocaine and PRP in DASH-E and VAS-P over time (Fig. 3a, b).

The estimated power calculated with our current data, for small, medium and big differences for DASH-E were 0.12, 0.26 and 0.93, respectively. Likewise, estimated powers for VAS-P were 0.18, 0.81 and 0.99, respectively.

### Safety

Twenty-three adverse events in twelve patients (six patients from each treatment group) were reported as probably (*n* = 22) or likely (*n* = 1) related to treatment. Pain and swelling were the most commonly reported in the 6 weeks following tenotomy. In addition, throughout the 12-month follow-up, a total of 593 events were reported as unlikely or not related to the treatment. They included headache, migraines, back pain, shoulder pain, gastrointestinal discomfort and allergies, with cold and flu being the most common among others.

## Discussion

Our hypothesis stated that PRP could be an effective adjuvant to elbow tenotomy (medial and lateral) in patients who were recalcitrant to conservative treatment. As tenotomy is an active treatment that per se influences outcomes in tendinopathy [10], our goal was not to test PRP on its own, but to determine the clinical efficacy of the combined procedure [tenotomy + PRP]. We found that two tenotomies (one strong, approximately 20–25 penetrations and bone abrasion, and the second weaker, approximately 10 penetrations) with PRP or lidocaine were effective to reduce pain and improve function over 6 months. Moreover, the improvement was maintained at 1 year after intervention, which is beyond the limits of recurrence. However, we did not find any clinical differences between the effects of the adjuvants, PRP and lidocaine, in pain reduction or in functional recovery at 6 or 12 months.

According to a recent meta-analysis [14] (including 12 studies of painful lateral epicondylopathy), an effect size of 0.57 favoured PRP treatment. Thus, our trial was underpowered, as a sample size of 73 patients per group would be necessary to detect any significant difference in clinical outcomes. According to these data, most studies in the literature, except one study [23] examining PRP in elbow epicondylopathy, are underpowered. Moreover, the probability of superiority and the number needed to treat varied depending on the comparator [24].

In fact, PRPs have been compared with various injectables, including autologous blood [25–27], saline [28–30] or corticosteroids [22, 28, 29, 31], using different treatment protocols. In addition to the choice of the comparator, the use (or not) of tenotomy or an alternative milder peppering technique (approximately five perforations) can yield to different temporal differences in PRP efficacy. For example, Peerbooms et al. [22] and Liebiedzinsky et al. [31] used a peppering technique and demonstrated that L-PRP was superior to corticosteroids at 12 months but not at the 3- and 6-month follow-ups, corroborating data from other studies with only a 3-month follow-up [28]. On the other hand, other studies with small samples per group (20–25 patients) failed to find differences between saline and L-PRP at three months [28] or between saline and pure PRP at 6 months or 12 months [30]. The latter study involved patients with recent epicondylitis. Likewise, when autologous blood was used as a comparator, there were no relevant differences in primary outcomes over a 6-month period [25, 26]. According to the estimated potency using our clinical data, our results are not conclusive due to an inadequate sample number. However, all these studies are valuable as they contribute to stronger findings through meta-analyses and umbrella reviews.

Although we failed to show differences between PRP and lidocaine, our results do match the promising results observed in a previous multicentre study comparing L-PRP with anaesthetics in lateral elbow tendinopathy [23]. Weak evidence (retrospective case-controlled study with few patients) for better outcomes with pure PRP at 6 and 12 months compared to bupivacaine has been reported [32]. In contrast, Palacio et al. [29] did not find any difference at 3 or 6 months when comparing PRP with anaesthetics or corticosteroids in untreated patients after blind 3 mL injections. To the best of our knowledge, only the three above referenced studies [23, 29, 32] compared injections of PRP versus anaesthetics. However, the number of treatments, PRP formulation and injected volume and intensity of tenotomy differed among studies.

We chose lidocaine as a comparator because it is our routine practice to enhance patient and physician comfort during the procedure. Indeed, tenotomy is very painful. Whether lidocaine is an active or placebo control in this context is controversial. Actually, there is general concern regarding the intratendinous administration of anaesthetics, because it might compromise cell viability [33]. Similarly, administering anaesthetics peripherally, which is common clinical practice in order to reduce pain during intervention, could damage cells from the extrinsic compartment that participate in early extrinsic repair [34]. Furthermore, the volume of lidocaine delivered in the peritendon of the common extensor origin varied between studies and was as high as 10–15 mL lidocaine (10 mg/mL) [28]. Lidocaine may induce cytotoxicity (apoptosis and/or necrosis) through mitochondrial insults mediated by intracellular radical oxygen species (ROS), by specific mitogen-activated protein kinases (MAPKs) (i.e., ERK1/2, JNK and p38) and by caspase-3/7 in vitro [34]. However, differences in cytotoxic vulnerability between healthy and tendinopathic cells in the intrinsic and extrinsic compartments and whether PRP reinforces lidocaine cytotoxicity or not have not been explored yet. Despite all these negative in vitro data related to lidocaine, our patients in the lidocaine group showed a similar safety profile to that of patients receiving PRP.

In addition to the choice of active (blood, corticosteroids) or placebo comparators (saline), the efficacy of PRP in tendon interventions may be strongly linked to other factors that can have biological/clinical relevance, e.g., first, whether the intervention is echo-guided to control the spatial delivery of the product, i.e., peri-tendinous, intra-tendinous or both, and second, the intensity of the tenotomy procedure, which can vary from 5 to 50 penetrations. Finally, the composition of PRPs varies between studies in terms of product composition and application protocol. According to the Platelet Physiology Subcommittee of the Scientific and Standardization Committee of the International Society on Thrombosis and Haemostasis (ISTH), the plasma product used in this trial was recalcified at 4.5 mL (× 2) PRP IIA1 [35]. The biological properties of this PRP product regarding inflammation and angiogenesis [11], cell migration, proliferation and anabolic effects have been fully examined in tenocyte cultures [12, 17], but they used frozen-thawed allogeneic PRP.

Compared to other clinical studies, we used a relatively high volume (4–5 mL versus 1–3 mL). This high volume implies diffusion of PRP away from the injection sites, reaching adjacent peritendinous tissues, including the subcutaneous fat and peritenon (epitenon) [36, 37]. Actually, activation of the extrinsic compartment (with a higher number of cells than the proper tendon) is also needed to drive healing through the complementary actions of intrinsic and extrinsic cells. However, how the extrinsic and intrinsic compartments interact in the failed healing response is unknown and whether PRP (or other regenerative products) should be delivered intra- or peritendinously or both and their interaction with local anaesthetics should be investigated. According to recent data [18], peritendinous delivery of PRP in enthesopathies was effective in pain reduction and produced better results than injections within the proper tendon in proper tendinopathies.

Our protocol also differed from those that published the number of interventions. In fact, consistent with the idea that tendinopathy is acquired through many years and the fact that a unique intervention is unlikely to change the tendon structure and fate of tendinopathy, we have performed two interventions (tenotomies). Other authors [25, 30, 38] repeated the injections 2–4 weeks apart. While there is no evidence on the benefits of the second injection in epicondylopathy [39], a second injection enhanced outcomes in a randomized prospective study in patellar tendinopathy [40]. Hopefully, in the future, interventions would be tailored to patient subgroups with different tendon needs.

Our study has several limitations. First, the diagnosis was merely clinical based on clinical signs and local pain. Second, whether PRP is superior to lidocaine in this context remains unsolved in this trial, in part because of the large amount of missing data not attributable to lost patients, but for other reasons. We did not perform telephone reminders regarding patient appointments and did not control if those patients were examined in the sonographic service attending their orthopaedist appointment. In fact, some of them missed their clinical appointment because of long waiting times. Moreover, some DASH-E questionnaires were incompletely filled and could not be computed.

Third, the lack of a dry tenotomy group in our trial means that we cannot, with certainty, rule out the effect of “dry” tenotomy as the main driver of therapeutic benefits. Moreover, the effects of PRP or lidocaine without tenotomy remain to be elucidated. Lastly, DASH-E is the sole functional score validated in Spanish, but it has important limitations for assessing elbow function, as other upper limb pathologies result in artefacts in DASH-E scores.

## Conclusions

The intention-to-treat analysis revealed that a double tenotomy, with lidocaine or PRP as adjuvants, is effective in producing a clinically significant reduction in DASH-E and VAS-P scores in a cohort of patients with recalcitrant elbow tendinopathy. In up to 70% of the cases, tenotomy can succeed when conservative therapies have already failed, and both adjuvants appear to be of comparable efficacy with this limited sample size. We thus recommend “humid” tenotomy with PRP/lidocaine as an important second line option in patients in whom conservative physical therapy has failed. As always, “the devil is in the details”; thus, we have focused here on describing and discussing the details of our protocol that could have clinical consequences.

## Abbreviations

BMI: Body mass index
CI: Confidence interval
DASH-E: Spanish version of the Disabilities of the Arm, Shoulder and Hand questionnaires
ECRB: Extensor carpi radialis brevis
HUC: Hospital Universitario Cruces
IQR: Interquartile range
ISTH: International Society on Thrombosis and Haemostasis
L-PRP: Leukocyte- rich, platelet-rich plasma
M: Mean
MAPKs: Mitogen-activated protein kinases
NA: Not applicable
NSAIDs: Non-steroidal anti-inflammatory drugs
PRP: Platelet-rich plasma
SD: Standard deviation
US: Ultrasound
VAS-P: Visual analogue scale for pain
